# Contributions of university activities to active aging: grounded theory*

**DOI:** 10.1590/1980-220X-REEUSP-2021-0237

**Published:** 2022-01-31

**Authors:** Flávia Maria Derhun, Giovana Aparecida de Souza Scolari, Leidyani Karina Rissardo, Maria Aparecida Salci, Montserrat Puig Llobet, Lígia Carreira

**Affiliations:** 1 Secretaria da Saúde do Estado do Paraná, Maringá, PR, Brazil.; 2 Universidade Estadual de Maringá, Maringá, PR, Brazil.; 3 Prefeitura de Maringá, Maringá, PR, Brazil.; 4 Universidade de Barcelona, Barcelona, Espanha.

**Keywords:** Aging, Aged, Public Policy, Learning, Universities, Community-Institutional Relations, Envejecimiento, Anciano, Política Pública, Aprendizaje, Universidades, Relaciones Comunidad-Institución, Envelhecimento, Idoso, Política Pública, Aprendizagem, Universidades, Relações Comunidade-Instituição

## Abstract

**Objective:**

To understand the contributions of university activities for active aging of elderly people committed to the Open University for the Third Age program.

**Method:**

This is a qualitative study guided by Symbolic Interactionism as a theoretical framework and by the Grounded Theory as a methodological framework. Data were collected between April and October 2020, through individual interviews with the elderly, coordinators, and professors of an Open University for the Third Age. The software *Atlas.ti* ^®^ was used to support the analysis, which took place through open, axial, and integration coding.

**Results:**

Participation in university activities contributed to strengthening the pillars that support the active aging policy. Contributions were driven by lifelong learning, included in the program through nonformal continuing education, which optimized opportunities for the elderly’s health, participation, and safety.

**Conclusion:**

The contributions arising from participation in university activities favor the (re)construction of the elderly’s resilience in coping with everyday situations.

## RESUMO

**Objetivo:**

Compreender as contribuições das atividades universitárias para o envelhecimento ativo de idosos vinculados à Universidade Aberta à Terceira Idade.

**Método:**

Estudo qualitativo orientado pelo Interacionismo Simbólico como referencial teórico e pela Teoria Fundamentada nos Dados como referencial metodológico. Os dados foram coletados entre abril e outubro de 2020, por meio de entrevistas individuais junto a idosos, coordenadores e docentes de uma Universidade Aberta à Terceira Idade. O *software Atlas.ti* ^®^ foi utilizado para apoio à análise, que ocorreu por meio da codificação aberta, axial e integração.

**Resultados:**

A participação nas atividades universitárias contribuiu para o fortalecimento dos pilares que sustentam a política do envelhecimento ativo. As contribuições foram impulsionadas pela aprendizagem ao longo da vida, incluída no programa por meio da educação permanente não formal, e que otimizou oportunidades para a saúde, participação e segurança dos idosos.

**Conclusão:**

As contribuições advindas da participação nas atividades universitárias favorecem a (re)construção da resiliência dos idosos no enfrentamento às situações cotidianas.

## RESUMEN

**Objetivo:**

Comprender las contribuciones de las actividades universitarias para el envejecimiento activo de ancianos vinculados a la Universidad Abierta a la Tercera Edad.

**Método:**

Estudio cualitativo orientado por el Interaccionismo Simbólico como referencial teórico y por la Teoría Fundamentada en los Datos como referencial metodológico. Los datos fueron recolectados de abril a octubre de 2020, por medio de entrevistas individuales con ancianos, coordinadores y docentes de una Universidad Abierta a la Tercera Edad. El *software Atlas.ti* ^®^ fue utilizado para dar apoyo al análisis, que ocurrió por medio de la codificación abierta, axial e integración.

**Resultados:**

La participación en las actividades universitarias contribuyó para el fortalecimiento de los pilares que sostienen la política del envejecimiento activo. Las contribuciones fueron impulsadas por el aprendizaje a lo largo de la vida, incluidas en el programa por medio de la educación permanente no formal y optimizaron oportunidades para la salud, participación y seguridad de los ancianos.

**Conclusión:**

Las contribuciones advenidas de la participación en las actividades universitarias favorecen la (re)construcción de la resiliencia de los ancianos al enfrentar las situaciones cotidianas.

## INTRODUCTION

The demographic transition has brought visibility to research including the elderly population. Risk factors for disability, morbidity, and mortality have been widely debated, but recently, approaches focusing on aging as a phase in which it is possible to enjoy well-being have emerged^(1–3)^. In this regard, almost two decades ago, the active aging policy was launched, the structure of which is centered on optimizing opportunities for health, participation, safety^(4–5)^, and lifelong learning^(5)^.

With regard to health, the policy considers that when risk factors for diseases and disabilities are few and protective factors are high, people tend to enjoy life with more quality. Regarding participation, it is considered that individuals, as they age, can and should continue to contribute to society through paid and unpaid activities, according to their wishes and capacities^(4–5)^.

The safety pillar states that policies and programs shall address the needs and rights of older people regarding social, physical, and financial protection^(4–5)^. Finally, lifelong learning indicates that access to information promotes well-being and is a way of equipping people to remain healthy and engaged in society^(5)^.

From this perspective, the aforementioned policy presents the challenges of global aging and highlights the need for government sectors to articulate themselves to finance the various demands, especially the social and health ones. The opportunities to be explored are addressed in the same way and are linked to changes in attitudes regarding aging, seeing it as a stage in the development of skills that help in managing daily challenges and also allow the elderly to contribute to society^(3–6)^.

Currently, there is no consensus on which activities are part of an active life^(7)^. However, studies have pointed out the engagement in social, physical, civic, labor^(8)^ and educational practices as an indicator of active aging^(2,7,9)^. Such activities have different goals, interactions during participation and, consequently, repercussions^(7)^.

For the individual, the contributions of active involvement depend on the level of effort, of required personal resources, and of the satisfaction generated when carrying out the activity^(2)^, while, for society, they are associated with the potential socioeconomic impact^(2,10–11)^. In this regard, special emphasis will be given here to educational activities that take place in the context of the Open Universities for the Third Age (*UNATI*).

Such programs are generally linked to universities and optimize their structural, professional, and financial resources to act^(12)^. UNATIs have been sought due to the nonformal nature of teaching and the need of the elderly population to occupy the available time, to expand social relations, and to acquire knowledge^(13)^. Repercussions of participation to the dimensions of active aging were reported in the literature^(1,14,15)^; however, there is no evidence that they are structured on the policy pillars and encompass both the contributions to the individual and to society.

When considering the particularities of such a scenario and the complexity of the elderly population requirements, which demand from services and professionals the capacity to give responses guided by public policies^(16)^, the question is: what are the contributions of the participation in UNATI to active aging? This study aimed to understand the contributions of university activities for the active aging of elderly people linked to UNATI.

## METHOD

### Design of Study

Qualitative approach study guided by the theoretical framework of Symbolic Interactionism (SI)^(17)^ and methodological framework of Grounded Theory (TFD) in the Straussian perspective^(18)^.

### Local

The study setting was a UNATI linked to a public university in southern Brazil. This university started its activities in 2010 charging no fee from the participants. It had 41 subjects distributed in six different thematic axes: art and culture; communicative processes and procedures; physical and mental health; physical and social environment; law and citizenship; humanities. The subjects were offered from Monday to Friday, in morning and afternoon shifts, with a workload varying according to the syllabus.

The selection of the local was made based on the facility of access to UNATI, since the researcher worked for three years as a volunteer professor in the program, offering a course on the Brazilian Public Health System (*SUS*). Participation in the discipline occurred because it had already been conducted by professors from the department to which the researcher was linked.

### Population and Selection Criteria

UNATI has approximately 400 students, 35 teachers, and 02 coordinators (general and pedagogical). Initially, elderly people approached for convenience were those meeting the inclusion criterion of attending courses at UNATI for at least six months. They were the first sample group.

Data collection and analysis of the first sample group provided important support; however, at a certain point, the new data no longer helped to understand the object of study and gaps were defined. An in-depth comprehension of the role of UNATI and its interface with other University initiatives was required.

Up to that moment, the analysis showed some aspects that influenced the possibilities for active aging in the scenario, such as the existence of partnerships with other services/departments of the University for the performance of teaching, research, and extension projects, the performance of events (seminars/ symposiums, etc.) via UNATI, the search/optimization of human resources to act in the activities inherent to the Program.

The significant relationships of such aspects with the work process and/or initiatives proposed by the UNATI Coordinations were noticeable and, therefore, the following questions arose: what is the view of UNATI’s Coordinations regarding active aging? How does UNATI’s Coordination work to promote active aging?

In this regard, the following hypothesis was formulated: the actions of UNATI’s coordinations have the potential to turn the expansion of possibilities (un)feasible for active aging in the university context. Data were then collected from the second sample group, composed of UNATI’s General Coordination and Pedagogical Coordination.

Data analysis of the second sample group revealed that the involvement and scope of institutional initiatives proposed by the Coordinations were influenced/limited by State policies for public universities. Furthermore, this group reinforced the heterogeneity among UNATI’s participants and the need for strategies to minimize the difficulties of engagement arising from it. In this regard, the role of teachers was constantly indicated as a modulator of actions and interactions within the scope of UNATI.

Interviews with this group provided relevant information, but other responses were needed to develop the properties and dimensions of the categories. For that, the hypothesis raised was: in addition to the initiatives proposed by the Coordinations, the development of actions of institutional competence and the teaching-learning approaches adopted by the teachers have the potential to turn the interaction between the elderly and the university community (un)feasible. For the teachers, who made up the third sample group, the following inclusion criterion was adopted: have been working at UNATI for at least six months.

The collection was concluded with the third sample group, respecting the theoretical saturation criteria^(18)^. At that time, the new information no longer changed the configuration of the phenomenon found, the categories appeared consistent, and the relationships between them were established.

### Data Collection and Analysis

Data were collected between April and October 2020, through individual interviews conducted by a doctoral student with experience in qualitative research. The interviews were guided by a semi-structured script, evaluated by three PhDs with expertise in qualitative research and/or gerontology. Participants were invited to participate via telephone contact.

There were no refusals to participate in the research and, also, there was no repetition of interviews, which had an average duration of 38 minutes and were mediated by technologies (video calls of WhatsApp^®^ and *Google Meet* and voice phone call). During the collection, only the researcher and the interviewees were present at the call. The audios were recorded on a digital device and transcribed.

The coding and analysis process was carried out by the main researcher and supported by the software ATLAS.ti^®^ version 8.4.24 and took place in three interdependent stages: open, axial, and integration^(18)^. The central phenomenon of the study was “UNATI as a way to optimize opportunities for active aging”.

The theoretical matrix was validated in a virtual meeting, in which representatives of the three sample groups studied took part, and the following criteria were used: adjustment, understanding, and theoretical generalization^(18)^. Due to the theoretical density, this text addresses the category “(Re)developing resilience from university activities for the elderly”, which, in the use of the paradigmatic model, is configured as consequences/results.

### Ethical Aspects

All ethical precepts in force for research involving human beings were followed, in accordance with Resolution 466/2012. Care with potential risks related to power relations was guided by Resolution 510/2016, so that, during the research process, the non-hierarchical researcher-participant relationship was built from dialogue and reflexivity.

The project was assessed and approved by the Ethics Committee of the university to which the researcher was committed, with opinion No. 4.010.03/2020 and CAAE:30056920.5.0000.0104. All participants were informed about the research and signed the Free Informed Consent Form, via electronic form. Participants were identified with the letters E, C and T, corresponding to the terms Elderly, Coordination, and Teacher, respectively, followed by Arabic numerals, according to the order in which the interviews were carried out.

## RESULTS

The study included 14 (fourteen) elderly people, 02 (two) coordinators, and 06 (six) teachers linked to UNATI. Regarding the elderly, nine were women, aged between 62 and 82 years, with most of them having higher education (n = 7) and high school (n = 3). Two had formal work activities; 11 were engaged in voluntary activities, and 11 reported at least one chronic condition. The duration of participation in UNATI was between one and ten years, with an average of four years.

Regarding the coordination and teachers, four were men, aged from 28 to 72 years old and an average of 50 years old. Three had a master’s degree, four a doctorate degree, and one a PhD degree. The length of work at the University was between 03 and 25 years, with an average of 15 years. The average of work at UNATI was six years, ranging between 02 and 11 years.

**Figure 1.**
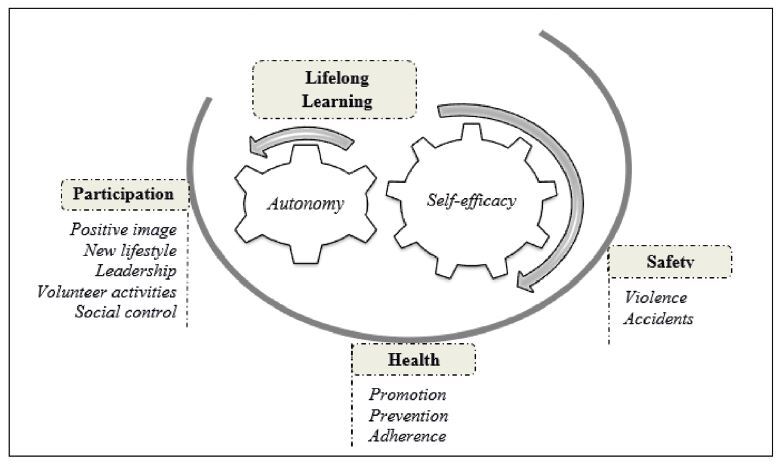
Diagram of the consequences component “(Re)developing resilience from university activities for the elderly”.

Below is the category “(Re)developing resilience from university activities for the elderly”, which lists UNATI’s contributions to active aging. Figure 1 demonstrates the subcategories and codes that make up the category, as well as the interrelationship between them.

**Chart 1.**
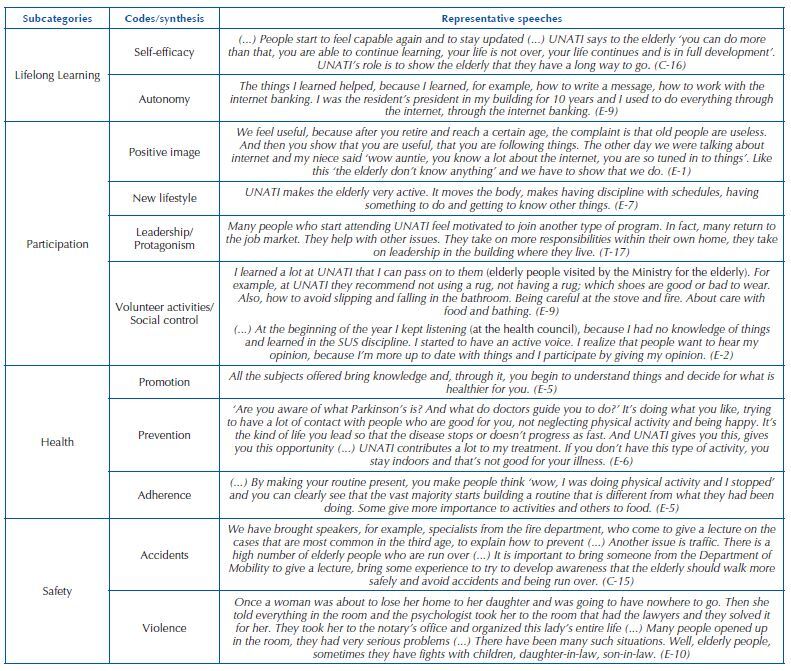
Distribution of subcategories, codes, and representative speeches of the research on the contributions of UNATI to the pillars of active aging – Maringá, Brazil, 2020.

Participation in UNATI contributed to the four pillars that support the active aging policy (health, participation, safety, and lifelong learning). Such implications proved to be interdependent and mutually reinforced; however, learning was obtained as the gear that awakened for the other pillars, as shown in Figure 1. The Chart 1 below shows the subcategories, codes, and representative speeches supporting the object of study.

With regard to contributions to the pillar of **lifelong learning**, the opportunity to learn and live together at UNATI, by itself, generated a feeling of appreciation in the elderly, who perceived themselves as subjects with potential for development. Engagement in intellectual activities allowed the elderly to feel empowered and capable, improved self-esteem and, consequently, expanded and/or developed the perception of self-efficacy, that is, the belief in their ability to perform the activities satisfactorily.

Interdependent on self-efficacy is the autonomy. Armed with knowledge and skills, the participants had a wider range of possibilities to assist in decisions and face everyday situations. Self-efficacy and autonomy were reinforced when there was recognition from the community, both internal and external to UNATI, about the elderly people’s ability to contribute to themselves and to their family and/or society.

Regarding the implications for the pillar of **participation**, engagement in the university activities showed a way to break negative stereotypes about the third age. The construction of a positive image about aging before the university community and society, especially to younger people, was noted. It was inferred that this is linked to the opportunity for the elderly, when participating in UNATI, to have a new lifestyle with a routine permeated by the adoption of personal practices aimed at individual and collective well-being. Such involvement also helped in periods of adaptation, such as retirement, moving to another city, and the loss of loved ones.

The (re)discovery of knowledge and skills provided by UNATI brought life purposes to the phase experienced. The elderly were able to take over the leadership and protagonism of their lives by participating in activities that are meaningful to them. The knowledge obtained strengthened the performance in instances that influence the rights of access to public actions and services (social and health), such as the social control, and also the performance of volunteer activities on different fronts. Finally, the most effective involvement in such areas created opportunities for the feeling of accomplishment and belonging to society.

With regard to contributions to the pillar of **health,** three aspects that support the subcategory were identified. The skills developed were configured as a way for the participants to expand the possibilities of action to improve their health. In this regard, health promotion was observed from the improvement of the participants’ personal and social resources, which led to empowerment to adopt a lifestyle suited to their needs.

Also in the field of promotion, it is worth highlighting the role of disciplines in the health area for functional health literacy. The formation of skills so that the elderly could obtain information related to health, understand them, and act based on them was noticed. It should be noted that this awakened and/or reinforced self-care actions, motivating and determining the adherence to behavioral measures. Knowledge for the proper use of medications, improved nutrition, access to the health system, physical activity, among others, was identified.

As for the prevention, the modification of risk factors and behaviors for chronic conditions make up the repercussions. Participation attenuated factors unfavorable to mental health and cognition (isolation, feeling of uselessness, lack of activities, etc.) and improved risk behaviors related to the development of various conditions and their complications, such as inadequate nutrition and physical inactivity. Contributions to well-being in situations of already established progressive diseases were also noted.

Considering the pillar of **safety,** actions of provision of knowledge and awareness in relation to the prevention of accidents (falls and traffic) and violence were identified. The actions took place based on the understanding that such problems are frequent in the elderly population. Furthermore, situations of financial violence perpetrated by family members were identified in the activities, and referrals to competent bodies were made.

UNATI’s contributions to the other three pillars of active aging (lifelong learning, participation, and health) constituted a foundation for the safety of the elderly by allowing them to be autonomous and independent. Finally, participation in UNATI gave the elderly the opportunity to develop a positive perspective on themselves and to carry out actions for their own, their family’s, and community’s well-being. This favored the (re)construction of the elderly’s resilience to deal with the situations imposed on them on a daily basis.

## DISCUSSION

The results allowed us to understand the contributions of participation in UNATI to the principles of active aging policy. The findings converge with its guidelines, in particular, with regard to the capacity of the activities provided in the context of UNATI to allow the elderly to perceive themselves as having the potential for their own well-being and engagement in society^(4–5)^.

Implications of program involvement for different dimensions of active aging have been reported by other studies. Participants have better self-assessment, behavior and responsibility for health when compared to nonparticipants^(1)^, as well as better level of physical activity, cognitive ability^(14)^, social life, and quality of life^(15)^. The present study advances by pointing out the contributions, in a more comprehensive way, based on the pillars that support the policy; and, by highlighting them, it points beyond the individual level, encompassing the potential implications for society.

A relevant point to trigger the repercussions listed in the study was self-efficacy, (re)developed through intellectual activities. It is seen as an internal resource or an individual’s belief about their ability to face daily challenges. Such beliefs, when strong, tend to motivate people to be more active to acquiring healthy behaviors^(19)^, while low beliefs are associated with the development of unfavorable conditions, such as mental disorders^(14)^.

Nonformal learning activities, when they occur in an inclusive and equitable manner, have shown an impact on the elderly’s well-being and ensured, even to the most vulnerable, a compensatory strategy to strengthen their autonomy and life satisfaction capacities^(6)^.

A study explored the learning outcomes acquired by different active aging activities and identified two types of orientation in relation to learning: self-focused and other-focused^(7)^ types. The former is related to self-knowledge and the acquisition of skills, resulting in improved self-confidence and self-esteem. The latter is linked to interpersonal knowledge, which improves the quality of relationships, as well as expands social networks. It is also linked to social knowledge, which encompasses the values of the community and its social systems^(7)^.

It is inferred that UNATI, by offering both types of knowledge through continuing education, provides opportunities for the elderly to act with confidence to meet their needs, undertake the search for the support network and/or services, when necessary, and act according to their wishes and capabilities in situations with the family and society. This indicates the support of contributions to the pillar of participation, that is, the construction of a positive image of aging, the adoption of a new lifestyle, leadership/protagonism and more effective engagement in activities.

In addition, lifelong learning at UNATI has involved the promotion of healthy lifestyles, since participation in the context has been recognized as a predictor of behaviors favorable to health, improving self-rated health^(1)^ and quality of life^(18)^. However, it is considered that engagement seems to have no effect on chronic conditions already installed, having implications for health through changes in behavior and access to health resources^(20)^.

With regard to safety, the offer of knowledge represented the actions taken. Furthermore, it is understood that the implications listed in the other pillars focus on the prevention of risk factors for accidents and violence. Examples of potentially minimized risk factors are the lack of social support, loneliness, dependence on everyday activities^(21–22)^, cognitive impairment, depression, chronic conditions^(21)^, the negative self-perception of health^(22–23)^, the state of frailty, and low/poor physical performance^(21–22)^.

Active aging goes beyond an individual responsibility^(10)^, and is a goal for federative entities to encourage, through public policies, the engagement of the elderly in significant tasks. The offer is necessary for people to have the opportunity to actively participate and contribute to individual, family, and community well-being. Al this to make demographic aging really a sustainable opportunity for social and health systems^(4,24)^.

Initiatives such as university activities are relevant. However, they have to further advance to expand access and to establish them as State policies and, if possible, financed, so that they do not depend so heavily on the interests of temporary administrations. Studies like this one and movements by organized civil society can compose the means for such a search.

One limitation of the study is the performance of interviews mediated by technologies. This distanced the researcher and interviewees, which may have hindered the apprehension of expressions, especially the nonverbal ones. The study of a single institution can also be a limitation, since each UNATI is structured from the physical, professional, and financial resources of the university to which it is linked^(12)^. This, however, does not invalidate the findings, which evidenced that a public program, which optimizes university resources, has generated positive repercussions for participants and, indirectly, for society.

## CONCLUSION

Participation in university activities for the elderly contributed to active aging. When considering the pillars supporting the policy, lifelong learning allowed the development and/or improvement of self-efficacy and autonomy; for the pillar of participation, the development of a positive image about aging, a new lifestyle and leadership, as well as engagement in volunteer activities and social control were observed; for health, participation contributed through promotion, prevention, and adherence; for safety, in particular, it contributed through the prevention of accidents and violence.

Such contributions formed a basis for (re)constructing the elderly’s resilience. Through this, they managed to handle situations involving their daily life and health more satisfactorily, allowing them to meet the needs of aging. The study has implications for practice as it reinforces the importance of academic management acting in the inseparability of teaching, research, and extension. Future research may explore how innovative teaching practices enhance the repercussions of participation in UNATI.
